# Selected Biochemical Markers Change after Oral Administration of Pesticide Mixtures in Honey Bees

**DOI:** 10.3390/toxics10100590

**Published:** 2022-10-05

**Authors:** Pawel Migdal, Agnieszka Murawska, Ewelina Berbeć, Mateusz Plotnik, Anita Skorus, Krzysztof Latarowski

**Affiliations:** 1 Department of Environment, Hygiene and Animal Welfare, Bee Division, The Wroclaw University of Environmental and Life Sciences, 25 C.K. Norwida St., 51-630 Wroclaw, Poland; 2 Department of Human Nutrition, The Wroclaw University of Environmental and Life Sciences, 25 C.K. Norwida St., 51-630 Wroclaw, Poland

**Keywords:** honey bee, *Apis mellifera*, pesticide compositions, insect physiology, biochemical markers

## Abstract

The honey bee is an important pollinator. In the environment, it can be exposed to many harmful factors, such as pesticides. Nowadays, attention is paid to evaluating the potentially harmful effects of these substances. This study aimed to evaluate the effect of worst-case environmental concentrations of pesticide mixtures on honey bee survival and selected physiological markers (the activity of ALT, AST, ALP, and GGTP, and the concentration of albumin, creatinine, urea, and uric acid). Pesticides of three different groups (insecticide—acetamiprid, herbicide—glyphosate, and fungicide—tebuconazole) and their mixtures were resolved in 50% (*w*/*v*) sucrose solution and given to bees *ad libitum*. After 24 h, hemolymph was collected. All mixtures caused higher mortality than single pesticides. Pesticides in mixtures caused disturbances in biochemical markers, and in some cases the interaction between pesticides was synergistic. The mixtures had individual effects on physiology, and the results were sensitive to changes in proportions.

## 1. Introduction

As a pollinator, the honey bee has a positive effect on increasing agricultural yields and preserving biodiversity. Bee pollination is valued at around 15 billion USD in the US, 19 billion USD in Europe, and 69 billion USD in East Asia [1]. For many years, the toxicity of pesticides to these insects has drawn the attention of researchers. One of the criteria for the division of pesticides concerns the type of target pest [2]. This classification includes, among others, insecticides, fungicides, and herbicides [3,4]. Honey bee contact with the pesticide at a sub-lethal dose may affect their behavior and/or physiology. Poisoning may occur after contact and/or oral exposure. Systemic pesticides, commonly used in developed countries, spread in plant tissues and may accumulate in plant nectar and pollen. In addition to exposure to harmful substances in the environment, bees may also come into contact with them in the hive, as they collect potentially contaminated nectar or pollen and store it in combs [5]. Due to the fact that many different pesticides are used in plant production, and their residues can accumulate in the environment, bees come into contact with many different toxins in various concentrations and proportions simultaneously [6]. When combined, an additive effect (i.e., the sum of the individual substances), synergistic effect (greater than an additive effect), or antagonistic effect (less than an additive effect) may occur. According to Regulation (EC) No 1107/2009 of the European Parliament and of the Council of 21 October 2009 concerning the placing of plant protection products on the market and repealing Council Directives 79/117/EEC and 91/414/EEC, mixing pesticides is a legal action unless it is expressly prohibited on the label of the product.

So far, the exposure of honey bees to pesticides has been shown to affect bee motor activity, navigation, feeding, learning ability, and memory, to weaken the immune and reproductive systems, and to activate the body’s antioxidant and detoxification mechanisms [7,8,9,10,11,12]. In order to fight harmful substances, the honey bee organism has developed many defense mechanisms within their detoxification and antioxidant systems. The detoxification mechanisms of the bee body mainly include enzymes involved in the metabolism of toxins or the detoxification process, i.e., cytochrome P450 monooxygenase (P450), glutathione transferase (GST), carboxylesterase (COE), aspartate aminotransferase (AST), alkaline aminotransferase (ALT) (ALP), gamma-glutamyl transpeptidase (GGTP), and bilirubin [12,13]. The mechanisms of the antioxidant system are designed to remove free radicals from the body. Antioxidants give electrons to free radicals and, as a result, the possibility of oxidizing other components is blocked. Among antioxidants, enzymatic antioxidants, such as glutathione peroxidase (GPX), catalases (CAT), superoxide dismutase (SOD), and glucose-6-phosphate dehydrogenase (GP6D), can be distinguished from non-enzymatic antioxidants (e.g., albumin, creatinine, glutathione, uric acid, urea, and vitamins) [11,13]. Many studies have confirmed that insecticides change enzyme activity and the content of some key substances [11,12,14,15,16,17]. However, reference values have not been estimated and there is still a lack of information about the influence of pesticide mixtures.

Our research aimed to investigate how oral exposure to pesticide mixtures affects the activity of selected hemolymph enzymes and non-enzymatic antioxidants of worker honey bees, i.e., ALT, ALP, AST, GGTP, albumin, creatinine, uric acid, and urea.

## 2. Materials and Methods

### 2.1. Research Material—Honey Bee Workers

Honey bee (*Apis mellifera* carnica) colonies used for research were treated against Varroa destructor using amitraz fumigation four times at 4-day intervals (12.5 mg/tablet; Apiwarol^®^, Biowet, Pulawy, Poland) before starting the experiment. To monitor the number of Nosema spp. spores, the hemocytometer method was used (30 bees per hive in three repetitions). After 28 days from the last fumigation, we selected 3 frames with bee brood in 20 days of apian development. Next, we took the brood to the laboratory and incubated it at temperatures of 34 and 70% of relative humidity. After 24 h, we collected the bees. Worker bees were placed in wooden cages (20 cm × 15 cm × 7 cm), each containing 100 workers and two inner feeders with 50% (*w*/*v*) sucrose solution (Chempur^®^, Piekary Śląskie, Poland) ad libitum. The adaptation process lasted 24 h at a temperature of 25 °C ± 0.5 °C and relative humidity of 70% ± 5%. Caged bees were maintained in the incubator in the same conditions described above until being used for the experiment [18]. Two-day-old bees were used in the study, and the bees were divided into 14 groups. Dead bees were utilized by a special biohazard waste company.

### 2.2. Experimental Setup

**Figure 1:**
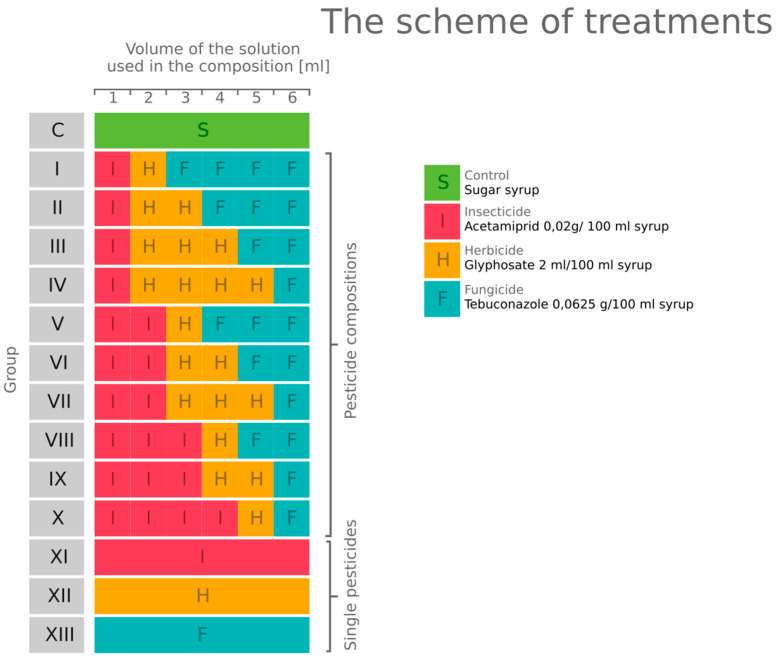
The scheme of treatments. Each group was fed *ad libitum* with a solution of a total volume of 6 mL. The solution composition differed between groups. The group abbreviated as C was a control group, fed with 50% (*w*/*v*) sucrose solution. Groups XI–XIII were fed with single pesticides dissolved in 50% (*w*/*v*) sucrose solution. Groups I-X were fed with pesticide compositions consisting of each type of pesticide in varying proportions.

Each group consisted of ten cages. The experiment was performed by feeding bees 50% (*w*/*v*) sucrose solution containing the established concentrations of particular pesticides for 24 h. Bees in the experimental groups were exposed to the single-pesticide commercial formulations or their trinary mixtures in different proportions. The control group was fed an untreated 50% (*w*/*v*) sucrose solution. The experiment used doses of pesticides recommended by the manufacturer for the selected active substance, which represented worst-case environmental concentrations. The bees were exposed to the insecticide Mospilan^®^ 20SP, Target, Kartoszyno, Poland (ai acetamiprid 20%), the herbicide Agrosar^®^ 360 SL, CIECH Sarzyna, Nowa Sarzyna, Poland (ai glyphosate 36%), and the fungicide Tebu^®^ EW, HELM, Hamburg, Germany (ai tebuconazole 25.8%) (Figure 1). Acetamiprid, as a cyano-substituted neonicotinoid, has a higher dose value that kills 50% of honey bees (LD50) compared to the nitro-substituted neonicotinoids (imidacloprid, clothianidin, thiamethoxam) and is considered less toxic than them [5,19]. However, its toxicity can be higher after being mixed with fungicide [20]. The LD_50_ of acetamiprid is 14,530 ng/bee (food exposure) and 8090 ng/bee (contact exposure) [5]. Mortality and syrup intake were recorded after 24 h.

### 2.3. Collection of Hemolymph

Hemolymph was taken from 100 alive worker honey bees from each group immediately after 24 h of oral exposure by removing the antennae with sterile tweezers. Hemolymph was conserved in a 20 μL end-to-end glass capillary without anticoagulant. Hemolymph from the control honey bees was collected at the same time. The test tubes were placed on the cooling block during the operation. After collecting the hemolymph, samples were stored at −80 °C [21].

### 2.4. Biochemical Analysis

Hemolymph biochemical parameters were determined using the Pentra 400 automated biochemical analyzer by Horiba ABX (Longjumeau, France). The colorimetric method with the use of bromocresol green and the creatinine kinetic method with alkaline picrate were used for measurement of complex formation coloring; urea—the UV enzyme test; uric acid—the enzymatic method using the Trinder reaction with Horiba ABX reagents were used to assess the level of albumin activity. Reagents from Randox (Crumlin, Great Britain) were used to assess the enzymatic activity (aspartate aminotransferase—AST, alkaline phosphatase—ALP, alanine aminotransferase—ALT, gamma-glutamyl transpeptidase—GGTP). Analysis kits for ALP—the photometric kinetic test in accordance with the recommendations of the International Federation of Clinical Chemistry (IFCC); for ALT and AST—the enzymatic method (UV detection) in accordance with IFCC recommendations; and for GGTP—the kinetic photometric test.

### 2.5. Statistical Analysis

Statistical analyses were performed using the R program, version 3.4.4 (R Core Team, 2018, R-3.4.4 for Windows, CRAN, Vienna, Austria), with the RStudio overlay, using, inter alia, the packages “dplyr”, “tidyr”, “agricolae”, and “ggplot2” [22]. The normality of the distribution was checked by the Shapiro–Wilk test, and the differences between the groups by the Kruskal–Wallis test with holm correction for multiple comparisons, α = 0.05. In Inkscape, the experiment scheme was made, and the charts were visually improved.

## 3. Results

### 3.1. Mortality and Syrup Intake

**Figure 2:**
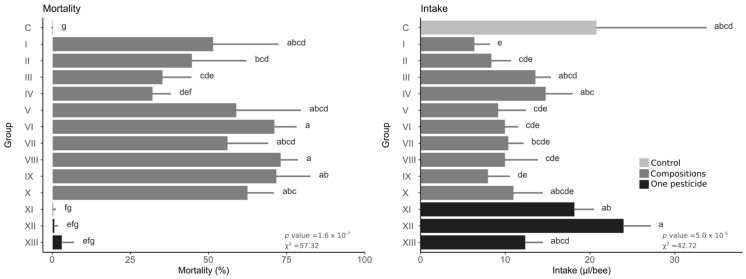
Mortality and syrup intake during 24 h of the experiment. Bars represent the mean, and error bars represent the standard deviation. The same letters between groups within one plot means no statistically significant differences (Kruskal–Wallis test with holm correction for multiple comparisons, α = 0.05); the statistical values are shown in the lower right corner of each graph.

In the control group, there was 0% mortality. Among the pesticides administered separately, none caused acute toxicity, with the highest mortality after fungicide administration being only 3.2% (Figure 2 and Table 1). All compositions showed mortality significantly higher than the control, while all except groups III and IV caused mortality higher than single pesticides. The highest mortality—over 70%—occurred in groups VIII, IX, and IV. Considering syrup intake within separately administered pesticides, the herbicide caused an intake similar to the control, while the fungicide intake was almost 50% lower, but the difference was not statistically significant. Mean values of syrup intake were lower in all compositions compared to the control, but the difference was statistically significant only in group I, with the lowest value over 3× lower than the control.

### 3.2. Enzymatic Activity

**Figure 3:**
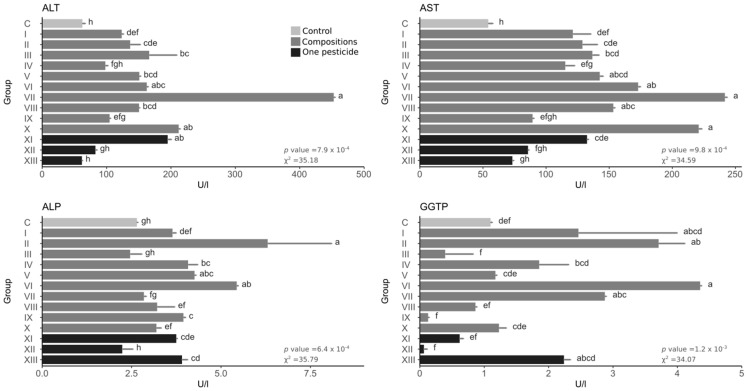
Enzymatic activity. Bars represent the mean, and error bars represent the standard deviation. The same letters between groups within one plot means no statistically significant differences (Kruskal–Wallis test with holm correction for multiple comparisons, α = 0.05); the statistical values are shown in the lower right corner of each graph.

The overall effect of pesticides was an increase in alanine aminotransferase (ALT). Of the single pesticides, only the insecticide increased ALT (Figure 3). All compositions caused an increase except group IV (with the highest amount of herbicide). The highest value was in group VII—more than 7× higher than the control and more than 2× higher than the single insecticide. Concerning aspartate aminotransferase (AST), the overall effect of pesticides was an increase in this parameter. Of the single pesticides, only the insecticide increased AST. All compositions except group IX caused an increase in AST. The highest increase occurred in group VII—more than 4× higher than the control and almost 2× higher than the insecticide, with a high value also in group X (composition with the highest proportion of insecticide). The overall effect of pesticides on alkaline phosphatase (ALP) was an increase in this parameter. Of the single pesticides, the insecticide and fungicide caused an increase in this parameter, while the herbicide had no effect. Most of the compositions increased ALP—only groups III and VII showed no increase. The highest value was found in group II—more than twice as high as the control, and a high value also in group VI. Generally, pesticides caused an increase or a decrease in gamma-glutamyl transpeptidase (GGTP), but the decreases were not statistically significant. Among the single pesticides, the differences were not significant in any, but the herbicide caused a very low GGTP value, while the fungicide caused an increase in GGTP. Among the compositions, groups IX and III caused a lower level of GGTP than in the control; however, the difference was not statistically significant. The highest value of GGTP occurred in group VI (approx. 4× higher than the control), and a significant increase in this indicator was also observed in groups II and VII.

### 3.3. Detoxification System Indicators

**Figure 4:**
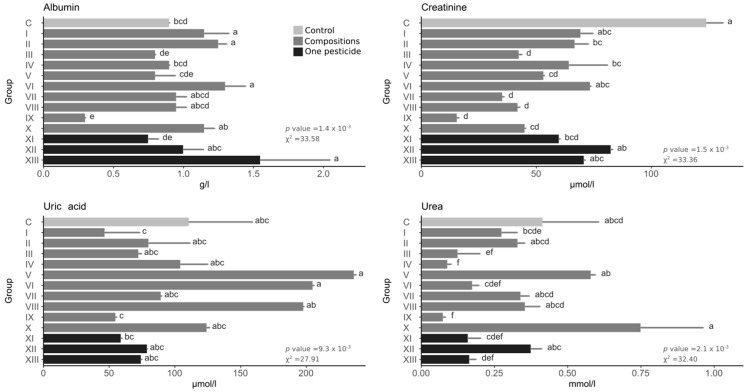
Detoxification system indicators. Bars represent the mean, and error bars represent the standard deviation. The same letters between groups within one plot mean no statistically significant differences (Kruskal–Wallis test with holm correction for multiple comparisons, α = 0.05); the statistical values are shown in the lower right corner of each graph.

Generally, pesticides caused the albumin levels to rise or fall. Among single pesticides, only the fungicide significantly changed the level of albumin, causing an increase (Figure 4). No composition had a higher level than the fungicide, while the highest values occurred in groups VI, II, and I. In the IX group, the albumin level was 3× lower than in the control. Pesticides generally caused a drop in creatinine levels. Among the pure pesticides, a statistically significant effect occurred only for the insecticide. Of the compositions, only two groups did not cause a statistically significant decrease (I and IV). The lowest value occurred in group IX, while low levels of creatinine also occurred in groups VII, VIII, III, and X. The values of urea acid after pesticide exposure were higher or lower than the control. Neither group differed statistically significantly from the control. The highest values were found in group V (2× higher than the control), and high levels of uric acid were also in groups VI and VIII. The lowest values were in groups I and IX and the single insecticide. Pesticides caused both an increase and a decrease in urea levels, with the higher values not statistically different from the control. The lowest value occurred in group IX (approx. 4× lower than the control), and low urea rates also occurred in groups IV and III.

## 4. Discussion

The pesticide compositions induced much higher acute toxicity than single pesticides. The differences were so large that this relationship can be called synergistic for all tested compositions. The synergistic mortality-increasing effect of the combinations of different types of pesticides has been previously observed [14,17,23]. This study suggests that the use of Acetamiprid, Glyphosate, and Tebuconazole in mixtures can significantly increase bee mortality. The specified toxicity in the first 24 h of our study was high. A bee in the environment, had it come into contact with such a set of pesticides, would not have returned to the hive. The concentrations of the pesticides did not exceed the manufacturer’s recommendations and were given to the bees separately; they did not show a significant effect on survival with short-term 24-h exposure. Walker et al. [24], investigating the linkage of an insecticide, fungicide, and adjuvant, had similar observations. The pesticides in combinations caused higher mortality than those used alone in the studies by Belsky et al. [25]. This shows that it is very important to compare the honey bee toxicity of individual pesticides and their mixtures. Additionally, the high toxicity of the pesticide compositions was confirmed by the syrup intake generally being lower in groups fed with the pesticide compositions than in groups fed with the single pesticides and control. Higher mortality was observed in the groups with pesticide mixtures despite lower syrup intake.

The pesticide mixtures had an individual effect; for each enzyme tested, there were compositions whose effect was greater than that of single pesticides. The general effect of single pesticides and mixtures on AST, ALT, and ALP was, if one occurred, an increase in these indicators. The observed effect on GGTP was either the increase or decrease of its activity compared to the control. Our research showed statistically significant changes in the activity of ALT, AST, and ALP compared to the control group in the case of most of the mixtures of pesticides. Similar observations of changes in activity were observed in the studies by Zhu et al. [17] when combining two insecticides containing the active substances imidacloprid and oxamyl, which resulted in a decrease in the activity of phenoloxidase—an immunity enzyme—and this effect did not occur when these substances were administered separately. Two binary compositions, thiamethoxam + λ-cyhalothrin and thiamethoxam + abamectin, caused a significant decrease in the activity and expression of a group of key insect detoxifying enzymes—glutathione S-transferases. A significant increase in mortality was also observed in these groups compared to the effects of single pesticides. In addition, thiamethoxam + abamectin caused a significant increase in ALP expression with a simultaneous decrease in the activity of this enzyme [23]. Changes in the activity of AST, ALT, and ALP have also been demonstrated in studies on the effect of imidacloprid [12]. In our research, the effect of selected plant protection products was an increase in the activity of these enzymes. Changes in the activity of these enzymes were also observed when bees were exposed to other substances. Bromfenvinphos, which is a substance used to treat bees during the infestation of the Varroa destructor mite, caused a decrease in the activity of AST, ALT, and ALP [26]. A similar effect was observed with the antifungal antibiotic amphotericin B [27]. During long-term coenzyme Q10 supplementation, an increase in ALT, AST, and ALP activity was observed [28]. Caffeine also caused an increase in the activity of these enzymes, and a similar effect was observed for piperine [29] and curcumin [30]. Increased concentrations of enzymes in the hemolymph may indicate a greater need for them by the organism.

Pesticides and mixtures caused a decrease in creatinine concentration, while in the case of albumin, urea, and uric acid an increase was observed in some groups, while a decrease was observed in others. Some mixtures of pesticides had a greater effect than individual pesticides; thus, in the case of the detoxification system, the individual effect of the mixture was also visible. The single use of the fungicide and some mixtures increased the concentration of albumin, and a similar effect was observed with the administration of bromfenvinphos used in the treatment of varroosis. Bromfenvinphos also caused a decrease in creatinine, urea, and uric acid levels [31]. Long-term administration of coenzyme Q10 caused a decrease in each of these indicators [28]. Caffeine supplementation caused an increase in uric acid and creatinine concentration and a decrease in albumin and urea concentration, and curcumin had a similar effect [30]. Albumin, creatinine, uric acid, and urea are substances that can also be classified as non-enzymatic antioxidants [28], hence they participate in the detoxification of oxidative stress agents.

## 5. Conclusions

Assessing the degree of pesticide effects on the honey bee in different combinations of substances continuously provides new information. It can be seen that the multidirectional exposure of bees to pesticides contributes to an increase in mortality and the disruption of the activity of biochemical markers. Such disturbances in the functioning of the organism may cause higher sensitivity to external factors. Showing the effects of single substances and comparing them with the effects of their mixtures is the basis for developing this research area.

## Figures and Tables

**Table 1:** The dose of each active ingredient per individual bee used in the research.

Group	Acetamiprid (µg)	Glyphosate (µL)	Tebuconazole (µg)
**C**	0.00	0.00	0.00
**I**	0.21	0.02	2.67
**II**	0.28	0.06	2.63
**III**	0.45	0.14	2.83
**IV**	0.49	0.20	1.54
**V**	0.61	0.03	2.88
**VI**	0.67	0.07	2.08
**VII**	0.69	0.10	1.08
**VIII**	1.00	0.03	2.08
**IX**	0.80	0.05	0.83
**X**	1.47	0.04	1.15
**XI**	3.64	0.00	0.00
**XII**	0.00	0.48	0.00
**XIII**	0.00	0.00	7.75

## Data Availability

The datasets generated and/or analyzed during the current study are available from the corresponding author on reasonable request.
